# Structure-guided engineering of a gatekeeper residue enhances polylactic acid depolymerization by a cutinase from *Papiliotrema nemorosa*

**DOI:** 10.3389/fbioe.2026.1936157

**Published:** 2026-09-10

**Authors:** Cheng Lin, Chunwang Li, Hanzhong Liang, Kexin Sun, Bin Song, Zhanyong Wang

**Affiliations:** College of Bioscience and Biotechnology, Shenyang Agricultural University, Shenyang, China

**Keywords:** cutinase, enzymatic degradation, interfacial adsorption, molecular dynamics simulation, polylactic acid, protein engineering

## Abstract

Enzymatic recycling of polylactic acid (PLA) is constrained by limited enzyme–polymer interactions, particularly insufficient adsorption onto highly hydrophobic solid surfaces. In this study, a cutinase-like enzyme from *Papiliotrema nemorosa* was heterologously expressed and characterized. Structural analysis suggested that Ser142 may function as a potential hydrophilic gatekeeper residue at the entrance of the substrate-binding pocket. Guided by this insight, computational screening and virtual saturation mutagenesis were employed to generate eight variants aimed at modulating enzyme–polymer interfacial recognition. Among these variants, the S142F variant exhibited the highest PLA-degrading capability. In addition to showing a 121.2% increase in PLA film adsorption capacity, S142F displayed significantly enhanced intrinsic catalytic turnover, resulting in an approximately two-fold enhancement in degradation efficiency, achieving 30.25% weight loss within 36 h at 60 °C. Furthermore, biochemical quantification revealed an approximately 1.58-fold increase in L-lactic acid release compared with the wild-type enzyme, providing additional biochemical support for the enhanced degradation performance. Molecular dynamics simulations suggested that the S142F substitution increased local hydrophobicity at the pocket entrance, which may strengthen enzyme–substrate interactions and contribute to improved substrate recognition. These findings suggest that structure-guided engineering of substrate-binding regions represents a promising strategy for regulating enzyme–polymer interfacial interactions and developing enhanced biocatalysts for PLA recycling.

## 1 Introduction

Global plastic production has exceeded 400 million tons annually, whereas less than 10% of plastic waste is effectively recycled (Geyer et al., 2017; Singh and Walker, 2024) The majority of plastic waste is either landfilled or released into the environment, causing severe environmental pollution and posing potential risks to marine ecosystems, soil environments, and public health (Amobonye et al., 2021). To mitigate the environmental burden associated with conventional plastics, biodegradable polymers derived from renewable biomass have emerged as promising alternatives to conventional petroleum-based plastics (Wang et al., 2020). Among them, polylactic acid (PLA) is one of the most widely used biodegradable polyesters due to its excellent biocompatibility, mechanical properties, and processability (Farah et al., 2016). However, PLA degrades very slowly in natural environments and may remain stable for extended periods (Karamanlioglu et al., 2017). Industrial composting at 55 °C–60 °C is typically required for efficient degradation, a temperature range close to the glass transition temperature (*T*_g_) of PLA (∼60 °C), where increased polymer chain mobility enhances ester bond accessibility (Lim et al., 2008; Brdlík et al., 2021). However, the practical degradation efficiency of PLA in real environmental and composting systems remains limited by its high crystallinity and hydrophobic surface characteristics, which restrict enzymatic accessibility.

Because spontaneous PLA degradation is slow under ambient conditions, enzymatic degradation has emerged as a promising, environmentally friendly, and controllable approach for PLA waste treatment and closed-loop recycling, owing to its high substrate specificity, mild reaction conditions, and low energy consumption (Wei et al., 2020; Xu et al., 2023; Shalem et al., 2024). Various enzymes, including lipases, proteases, and cutinases, as well as diverse whole-cell microbial consortia such as *Trichoderma* species, have been reported to degrade or induce structural changes in various polyester matrices such as PLA, polyethylene terephthalate (PET), and poly (butylene succinate) (PBS) (Hajighasemi et al., 2016; Hu et al., 2016; Dąbrowska et al., 2021). Cutinases and related polyesterases are particularly attractive because they possess a solvent-exposed active site, allowing direct interaction with both soluble and insoluble polyester substrates (Ribitsch et al., 2012; Austin et al., 2018; Joo et al., 2018). However, most wild-type cutinases exhibit limited degradation efficiency toward solid PLA, which is often associated with insufficient interfacial adsorption on the hydrophobic polymer surface and restricted access of the enzyme to ester bonds within the polymer matrix (Ribitsch et al., 2013). Therefore, improving enzyme–polymer interfacial recognition is an important direction for enhancing solid PLA degradation.

Numerous protein engineering studies have been conducted to improve cutinase performance, primarily focusing on enhancing thermostability and intrinsic catalytic efficiency. For example, Tournier et al. (2020) introduced an engineered disulfide bridge together with active-site mutations into the leaf-branch compost cutinase (LCC), significantly improving both thermal stability and polyester depolymerization activity. Although such advanced rational engineering has been predominantly demonstrated in PET-degrading systems, PLA and PET share several physicochemical challenges as crystalline and hydrophobic solid polyesters, including limited enzyme accessibility and interfacial interactions. Therefore, engineering strategies established for PET provide valuable mechanistic insights and design principles for improving PLA degradation. In particular, degradation of insoluble polymer substrates requires enzymes to first establish effective interactions with the polymer surface before ester-bond hydrolysis can occur. Compared with engineering efforts directed toward thermostability and catalytic turnover, enzyme–polymer interfacial recognition has received relatively limited attention. Engineering substrate-binding regions, particularly residues located at the entrance of the binding pocket, therefore represents a promising strategy. These gatekeeper residues are uniquely positioned to serve a dual function. They mediate macroscopic interactions between the enzyme and the surface while simultaneously governing microscopic substrate entry and active-site accommodation. For instance, Su et al. (2020) modified gatekeeper residues in *Humicola insolens* cutinase to reduce steric hindrance and enhance the hydrolysis of soluble polyacrylates. However, the structural determinants governing enzyme adsorption and substrate recognition on solid, hydrophobic polyester matrices such as PLA remains poorly understood, and whether pocket-entrance residues can be rationally engineered to enhance these interactions has yet to be fully explored.

Cr14CLE is a recently identified cutinase-like enzyme originally reported from *Cryptococcus nemorosus*. Arunrattanamook et al. (2023) reported that Cr14CLE can degrade PLA under mild conditions, with optimal activity at approximately 30 °C–40 °C and stable activity across pH 6–8. In this study, this enzyme was selected as the parental candidate for molecular engineering to improve PLA degradation. Because the source organism has been taxonomically reclassified as *P. nemorosa*, the enzyme described in the original literature is hereafter referred to as Cr14CLE when discussing the previous study. To establish a clear distinction, the codon-optimized recombinant enzyme heterologously expressed in *Escherichia coli* in this study is designated as PnCLE (*Papiliotrema nemorosa* Cutinase-Like Enzyme). Guided by structural modeling and substrate-binding pocket analysis, surface-exposed residues surrounding the putative substrate-binding pocket were systematically evaluated through a two-step computational screening strategy. Computational alanine scanning was first used to identify structurally tolerant candidate positions, followed by virtual saturation mutagenesis to identify substitutions with favorable predicted energetic effects and structural compatibility. The resulting candidates were then subjected to experimental validation, followed by molecular dynamics simulations to investigate their possible effects on enzyme–substrate interactions. This work highlights the potential of modulating enzyme–polymer interfacial interactions, rather than focusing solely on catalytic-center optimization, as a strategy for improving PLA-degrading cutinases.

## 2 Materials and methods

### 2.1 Gene, plasmids, and reagents

The amino acid sequence of *P. nemorosa* TBRC2959 cutinase-like protein (Cr14CLE, GenBank accession number: OR146962) was reverse-translated, codon-optimized for *E. coli* expression, and synthesized by Nanjing Zhongding Biotechnology (Nanjing, China). The taxonomic classification of the strain was verified according to NCBI GenBank annotation to ensure sequence consistency. The synthesized gene was cloned into a pET-22b (+) vector between the *Nco*I and *Xho*I restriction sites with a C-terminal 6×His tag. PLA (REVODE101, *M*_w_ = 12.9 × 10^4^, typical *T*_g_ = 56 °C–61 °C, *T*_m_ < 155 °C) was supplied by Zhejiang Hisun Biomaterials (Taizhou, China). All *p*-nitrophenyl (*p*-NP) esters were obtained from Shanghai Aladdin Biochemical Technology (Shanghai, China). All other reagents were of analytical grade unless otherwise stated.

### 2.2 Rational design and site-directed mutagenesis

The three-dimensional structure of PnCLE was predicted using AlphaFold 3 with default parameters (Abramson et al., 2024). The pLDDT scores were used for model confidence evaluation, and residues with pLDDT >95 were considered highly reliable for structural analysis. Based on the structural similarity with previously characterized cutinases, a monomeric model was used for subsequent structural analyses and computational simulations. Molecular docking of the L-lactic acid tetramer (LA_4_) was performed using AutoDock Vina 2.1.6. Computational alanine scanning and virtual saturation mutagenesis were conducted using FoldX (version 5.0). Prior to mutagenesis, the structure was energy-minimized using the RepairPDB module to remove steric clashes. Initially, computational alanine scanning was performed on 17 surface residues surrounding the putative substrate-binding pocket to evaluate their mutational tolerance. This computational analysis was used as a pre-screening step rather than experimental alanine mutagenesis. Residues showing neutral or stabilizing energetic effects upon alanine substitution were selected as target sites. Based on this tolerance analysis, residues 117, 141, 142, and 145 were selected for subsequent virtual saturation mutagenesis. Subsequently, the PositionScan module in FoldX was utilized to systematically mutate these selected tolerant residues to all 19 other amino acids, to evaluate whether bulky or hydrophobic substitutions could influence enzyme–polymer interfacial interactions. The energetic effects of mutations were classified as follows: ΔΔG < −0.5 kcal·mol-1 (beneficial), −0.5 ≤ ΔΔG ≤0.5 kcal·mol-1 (neutral), and ΔΔG >0.5 kcal·mol-1 (detrimental). A threshold of ΔΔG < −0.5 kcal·mol-1 was used for candidate selection. This threshold was selected to prioritize mutations with favorable predicted energetic effects and structural compatibility. Site-directed mutagenesis was performed using the QuickMutation™ kit (Beyotime, Shanghai, China) according to the manufacturer’s instructions. All mutations were confirmed by Sanger sequencing. Primer sequences and PCR conditions are provided in Supplementary Table S1, S2.

### 2.3 Protein expression and purification

Recombinant plasmids encoding wild-type (WT) and mutant PnCLE were transformed into chemically competent *E. coli* BL21 (DE3) cells. Positive transformants were selected on Luria–Bertani (LB) agar plates containing 100 μg mL^-1^ ampicillin and further confirmed by colony PCR. A single confirmed colony was used for each expression experiment to ensure reproducibility. Cells were cultured in LB medium containing ampicillin (100 μg mL^-1^) at 37 °C and 200 rpm until optical density at 600 nm (OD_600_) reached 0.6–0.8. Protein expression was induced with 0.2 mM isopropyl-β-D-thiogalactopyranoside (IPTG) at 25 °C and 200 rpm for 14 h. Cells were harvested by centrifugation at 8,000 × g for 10 min at 4 °C, resuspended in binding buffer (20 mM phosphate buffer, 5 mM imidazole, 500 mM NaCl, pH 7.4), and disrupted by sonication on ice (60 W, 3 s on/6 s off, total 30 min). The supernatant was collected by centrifugation at 12,000 × g for 30 min at 4 °C, and target proteins were purified using His-tag Protein Purification beads (Beaver Biosciences, Suzhou, China). The purified enzyme was dialyzed against 20 mM phosphate buffer (pH 7.4) at 4 °C overnight. Protein purity was verified by sodium dodecyl sulfate-polyacrylamide gel electrophoresis (SDS-PAGE), and protein concentration was determined using a BCA Protein Assay Kit (Beyotime, Shanghai, China).

### 2.4 Cutinase activity measurement

Cutinase activity was measured using *p*-nitrophenyl hexanoate (*p*NPH) as the substrate following established methods for polyester hydrolases (Hajighasemi et al., 2018; Shin et al., 2024). Briefly, 20 μL of enzyme solution was mixed with 400 μL of 1 mM *p*NPH prepared in 20 mM phosphate buffer (pH 8.0) and incubated at 60 °C for 10 min. Absorbance was measured at 405 nm. A substrate blank without enzyme was used to correct background hydrolysis. All assays were performed in triplicate to ensure statistical reliability. For the initial mutant screening assays, relative activity (%) was normalized to the absolute activity of WT PnCLE (100%). For temperature and pH profiles, the maximum activity within each experimental group was defined as 100%. To determine the kinetic parameters, the initial reaction velocities of WT PnCLE and the S142F mutant were measured using varying concentrations of *p*NPH (ranging from 0.05 to 2.0 mM) under the standard assay conditions (pH 8.0, 60 °C). The kinetic constants (*K*_m_ and *V*_max_) were calculated by fitting the data to the Michaelis-Menten equation and further analyzed using Lineweaver-Burk double-reciprocal plots. The turnover number (*k*_cat_) was calculated based on the *V*_max_ and the exact enzyme concentration used in the assay.

### 2.5 PLA emulsion and film degradation assays

PLA emulsion (0.2%, w/v) was prepared by ultrasonic emulsification. PLA pellets (0.2 g) were dissolved in 10 mL dichloromethane. Then, 100 mL of 20 mM phosphate buffer (pH 8.0) containing 0.01% (w/v) Plysurf A210G (Daiichi Kougyou Seiyaku, Tokyo, Japan) was added. The mixture was sonicated at 350 W for 12 min and stirred at 70 °C for 1 h to remove solvent. Final volume was adjusted to 100 mL. For PLA emulsion degradation, 500 μL enzyme (0.6 mg mL^-1^) was mixed with 1,500 μL emulsion and incubated at 40 °C. Absorbance at 630 nm was measured every hour. A buffer-only control was performed by replacing the enzyme solution with an equal volume of 20 mM phosphate buffer under otherwise identical conditions.

For PLA film degradation, PLA films (8 mm × 8 mm × 0.3 mm) were incubated in 2 mL enzyme solution (0.6 mg mL^-1^) at 60 °C for 36 h. To maintain constant enzymatic activity, the reaction solution was refreshed every 6 h. After incubation, films were washed, dried at 60 °C to constant weight, and weighed. Weight loss was calculated using Equation 1.

$$R\left(\%\right)=\frac{M_{0}-M_{1}}{M_{0}}\times 100\%$$ (1)

where $M_{0}$ is the initial weight of the PLA film (mg), and $M_{1}$ is the final weight after degradation (mg).

Surface morphology on the surfaces of the PLA films treated with the enzyme was observed using a scanning electron microscope (SEM, Hitachi SU8010, Tokyo, Japan). To biochemically quantify the terminal degradation products, the concentration of released L-lactic acid in the reaction supernatant after 36 h of film degradation was determined. The experimental groups consisted of PLA films incubated with either WT PnCLE or the S142F mutant, while the control group consisted of PLA films incubated in PBS without enzymes under identical conditions. The quantification was performed using an L-lactic acid assay kit (Nanjing Jiancheng Bioengineering Institute, Nanjing, China).

### 2.6 PLA film interfacial adsorption assay

PLA films were pre-washed with anhydrous ethanol and vacuum-dried. Each film was incubated with 1 mL of enzyme solution (0.6 mg mL^-1^) at 40 °C for 30 min with shaking at 180 rpm. A control without PLA film was included. Residual protein concentration was measured using a BCA Protein Assay Kit. The protein adsorption capacity ( $C_{p}$ ) was calculated using Equation 2:

$$C_{p}\left(\%\right)=\frac{C_{c}-C_{s}}{C_{c}}\times 100\%$$ (2)

where $C_{c}$ is the protein concentration of the control group (mg·mL^-1^), and $C_{s}$ is the residual protein concentration of the experimental group after adsorption (mg·mL^-1^).

### 2.7 Molecular dynamics simulations and binding free energy analysis

Molecular dynamics (MD) simulations were performed using GROMACS 2022.3 with the Amber99SB-ILDN force field for proteins and the GAFF force field for LA_4_ ligand. The systems were solvated in TIP3P water boxes and neutralized with Na^+^ ions. Energy minimization was performed using the steepest descent method, followed by 100 ps NVT and NPT equilibration simulations. Production simulations were performed for 100 ns with a time step of 2 fs. Trajectory analysis was performed using built-in GROMACS tools, including root-mean-square deviation (RMSD), root-mean-square fluctuation (RMSF), radius of gyration (*R*_g_), and solvent-accessible surface area (SASA). Binding free energies were calculated using the MM/GBSA method (Genheden and Ryde, 2015). The computational results were used to provide structural insights into possible differences in enzyme–substrate interactions between WT and mutant systems. All simulations were performed under identical parameters to ensure comparability between WT and mutant systems.

### 2.8 Statistical analysis

All experiments were performed in triplicate. Data are presented as mean ± standard deviation (SD). Statistical significance was analyzed using Student’s t-test and one-way ANOVA followed by Tukey’s multiple comparison test, with P < 0.05 considered significant.

## 3 Results and discussion

### 3.1 Structure-guided rational design and mutant screening

**FIGURE 1:**
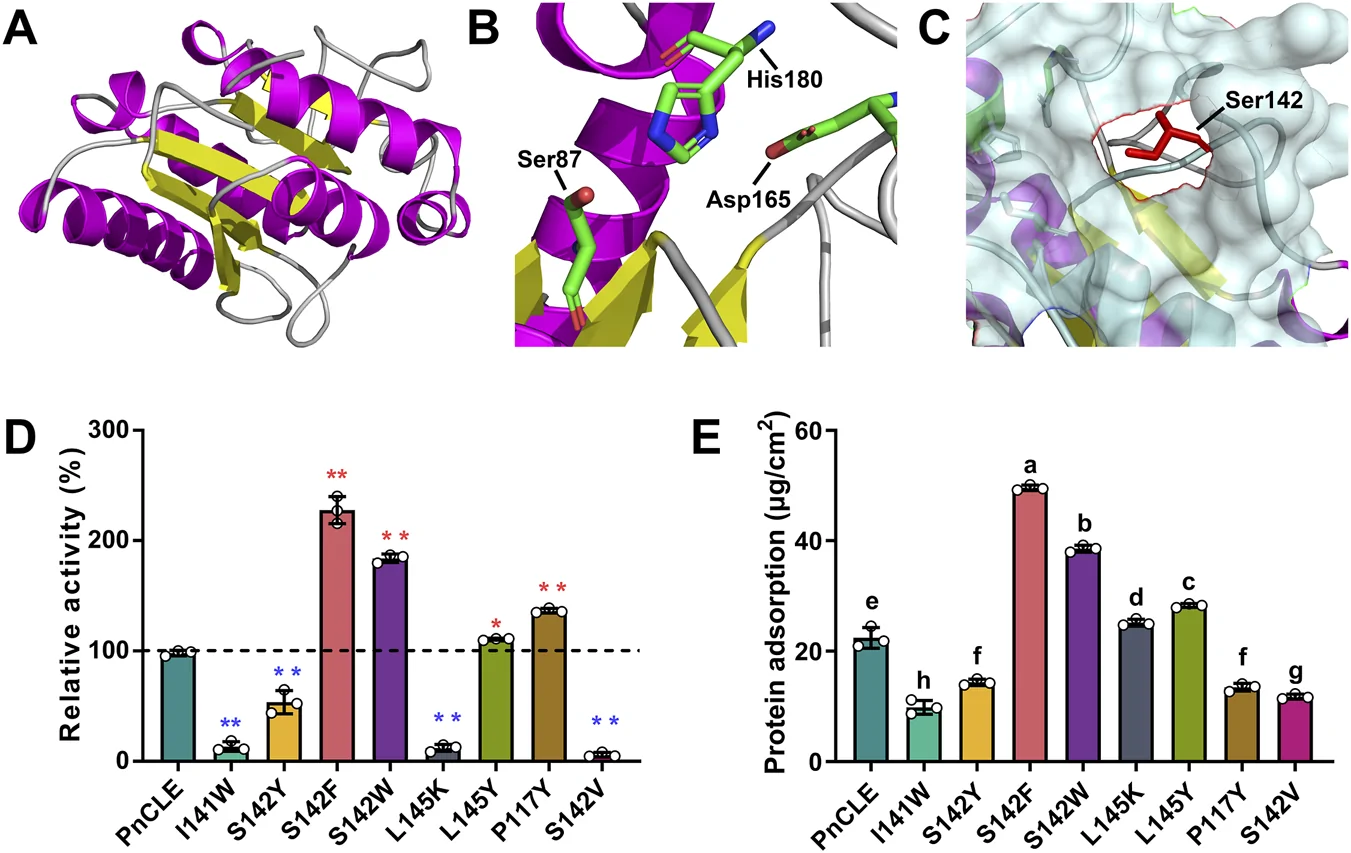
Structure-guided identification and experimental screening of PnCLE mutants. **(A)** Predicted three-dimensional structure of PnCLE. α-Helices, β-sheets, and loops are shown in magenta, yellow, and gray, respectively. **(B)** Close-up view of the catalytic triad of PnCLE, consisting of Ser87, Asp165, and His180. **(C)** Surface representation of the substrate-binding pocket of PnCLE, with Ser142 located at the pocket entrance. **(D)** Relative PLA emulsion degradation activity of WT-PnCLE and eight mutants. The activity of WT was defined as 100%. Red and blue asterisks indicate significantly increased and decreased activity compared with WT, respectively. Data are presented as the mean ± standard deviation of three independent experiments. (*p < 0.05; **p < 0.01). **(E)** Protein adsorption capacity of PnCLE and its mutants on PLA films after 30 min incubation. Different lowercase letters indicate significant differences (p < 0.05).

The recombinant PnCLE protein was expressed, purified, and verified in *E. coli* BL21 (DE3) (Supplementary Figure S1). Structural prediction using AlphaFold3 revealed that PnCLE adopts a typical α/β hydrolase fold (Figure 1A). The model exhibited a high confidence score, with an average pLDDT of 97.9 for the catalytic domain and pLDDT values exceeding 95 for residues within the active site pocket, indicating a reliable structural framework for subsequent analysis. Furthermore, the reliability of this model is supported by its high sequence identity (>70%) with the experimentally determined crystal structure of a homologous cutinase-like protein from *Cryptococcus* sp. (PDB ID: 2czq). The conserved α/β hydrolase fold and the catalytic residue arrangement between PnCLE and the homologous cutinase further support the reliability of the predicted structural model. Although the oligomeric state of PnCLE was not experimentally determined in this study, the extensive structural homology to previously characterized monomeric cutinases and cutinase-like enzymes (Masaki et al., 2005; Yang et al., 2023) supports the use of a monomeric configuration for downstream computational analyses. The catalytic triad, consisting of Ser87, Asp165, and His180, is located in the substrate-binding pocket (Figure 1B). Structural analysis further suggested that Ser142 is positioned at the entrance of the substrate-binding pocket, in close proximity to the putative substrate-binding region (Figure 1C). Therefore, Ser142 was considered one potential candidate involved in regulating substrate access and enzyme–polymer interfacial recognition. To avoid selecting engineering targets solely based on structural observation, residues surrounding the substrate-binding pocket were systematically evaluated using a two-step computational screening strategy.

Computational alanine scanning was initially performed using FoldX on 17 surface-exposed residues surrounding the substrate-binding pocket. This computational analysis served as a tolerance screening step rather than experimental alanine substitution, aiming to identify residues that could accommodate further engineering without compromising structural stability. The results indicated that substitutions at positions 117, 141, 142, and 145 were highly tolerated, showing neutral effects on global protein stability. These structurally tolerant residues were therefore selected as candidate engineering sites for subsequent virtual saturation mutagenesis using FoldX. Among these candidates, Ser142 was of particular interest because it is directly located at the entrance of the substrate-binding pocket and may contribute to substrate accessibility and polymer interfacial interactions.

Considering the hydrophobic nature of PLA, substitution of the hydrophilic Ser142 with more hydrophobic residues was hypothesized to enhance local enzyme–polymer interactions and potentially improve substrate binding and catalytic performance. Using a stringent stability threshold (ΔΔG < −0.5 kcal mol^-1^), together with structural compatibility and local hydrophobicity considerations, eight candidate mutants (P117Y, I141W, S142W, S142Y, S142F, S142V, L145K, and L145Y) were identified and selected for experimental validation (Supplementary Table S3, S4). These variants were subsequently expressed, purified, and evaluated for PLA emulsion degradation activity and interfacial adsorption capacity.

Initial screening assays, including PLA emulsion degradation and interfacial adsorption measurements, were conducted at 40 °C, based on the previously reported optimal temperature of the Cr14CLE (Arunrattanamook et al., 2023). As shown in Figure 1D, mutation effects varied substantially. Among all eight variants, S142F exhibited the highest relative activity in the PLA emulsion assay (∼232% of WT), followed by S142W (∼187%), P117Y (∼139%), and L145Y (∼113%), whereas I141W, S142Y, L145K, and S142V showed significantly reduced activity (below 50% of WT). These results indicate that substitutions at or near position 142 strongly influence the catalytic efficiency of PnCLE toward PLA emulsions. The time-dependent variation in PLA emulsion turbidity for all mutants is presented in Supplementary Figure S2. Interfacial adsorption assays further demonstrated that mutations also affected protein binding to PLA films (Figure 1E). S142F exhibited the highest adsorption capacity (∼49.6 μg cm^-2^), corresponding to a 121.2% increase compared with WT (∼22.4 μg cm^-2^), followed by S142W (∼38.6 μg cm^-2^, +73%), L145Y (∼28.3 μg cm^-2^, +24.5%), and L145K (∼25.2 μg cm^-2^, +12.3%). The remaining variants, including P117Y, I141W, S142Y, and S142V, exhibited lower adsorption capacities than WT. Notably, S142F simultaneously enhanced catalytic activity and surface adsorption, suggesting that improved interfacial interactions may contribute to enhanced PLA degradation performance.

### 3.2 Enzymatic properties of S142F mutant

**FIGURE 2:**
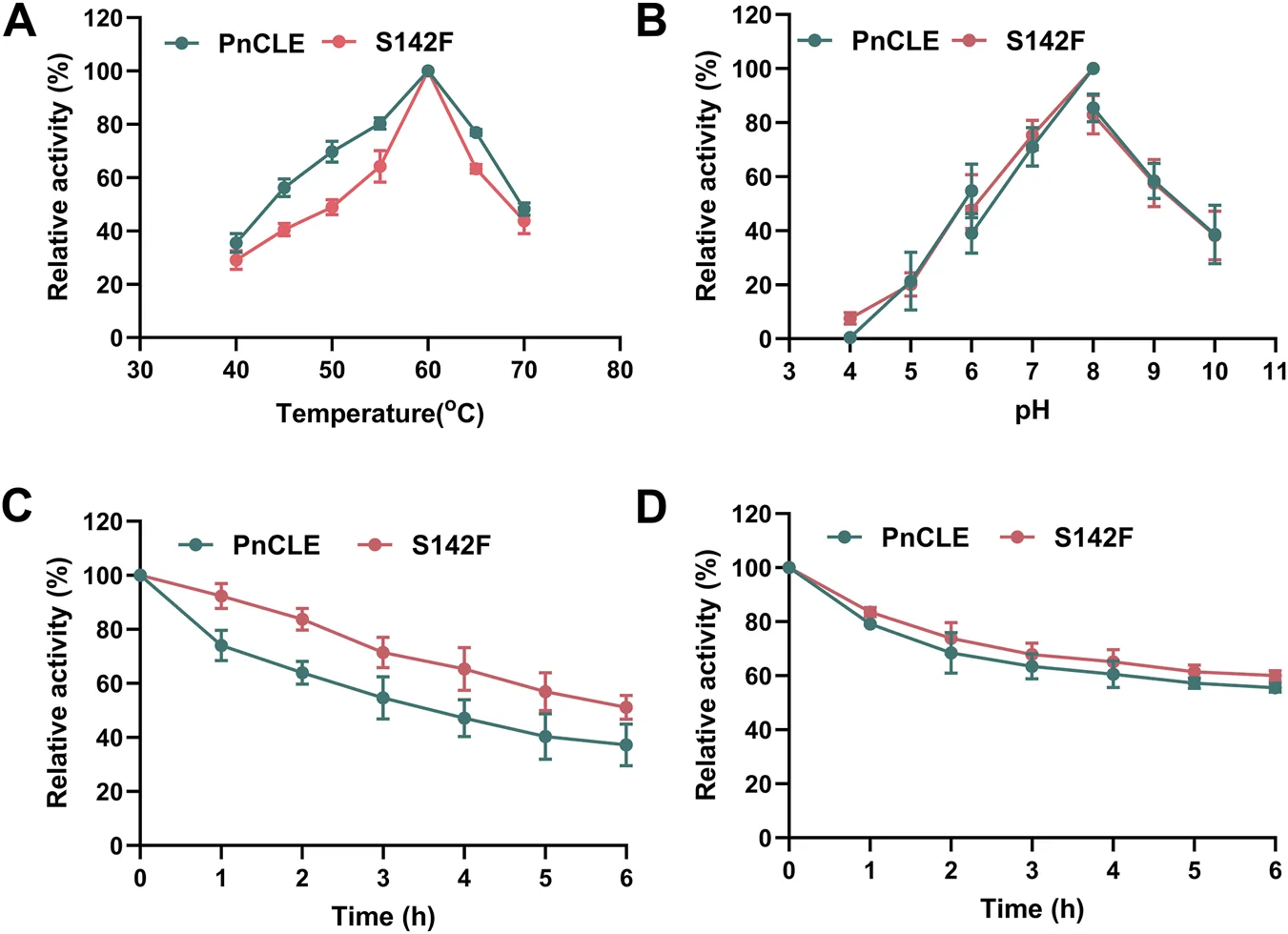
Enzymatic properties of WT PnCLE and S142F. **(A)** Effect of temperature on relative activity. **(B)** Effect of pH on relative activity. **(C)** Thermostability at 60 °C. **(D)** Stability at pH 8.0. The maximum activity of each experimental group was defined as 100% for its respective profile. The normalized values represent temperature- and pH-dependent activity profiles and should not be interpreted as direct comparisons of absolute activity between variants.

The enzymatic properties of the S142F mutant were characterized. Both WT PnCLE and S142F exhibited an optimal temperature of 60 °C (Figure 2A). This observed optimal temperature is higher than the 30 °C–40 °C reported for Cr14CLE (Arunrattanamook et al., 2023). Given that PnCLE and Cr14CLE share an identical amino acid sequence and were both heterologously expressed in *E. coli*, this discrepancy is unlikely to arise from sequence variation or host-specific expression effects. Instead, it may reflect differences in assay conditions, including substrate type and morphology, enzyme concentration, reaction time, buffer composition, and activity measurement methods. Therefore, the 60 °C value should be regarded as an apparent optimal temperature under the present experimental conditions. A direct side-by-side comparison under strictly identical conditions would be required to determine intrinsic thermal differences. Nevertheless, the high activity of PnCLE at 60 °C is particularly relevant, as this temperature closely matches both industrial composting conditions and the glass transition temperature (*T*_g_) of PLA (Mouhoubi et al., 2022), enhancing the practical applicability of the enzyme (Tokiwa and Calabia, 2006; Naser et al., 2021). The optimal pH for both enzymes was 8.0 (Figure 2B), and relatively high activity was maintained between pH 7.0 and 9.0, indicating a preference for neutral to weakly alkaline conditions. This pH profile may be advantageous for PLA hydrolysis systems (Shah et al., 2008), although the effects of acidic degradation products such as lactic acid require further investigation. Thermostability assays showed that the S142F mutant displayed higher residual activity than wild-type PnCLE during incubation at 60 °C (Figure 2C). After 6 h of incubation, the S142F mutant retained 51.1% of its initial activity, while wild-type PnCLE retained 37.3%. This result indicates that the S142F mutation moderately improved the thermal stability of PnCLE under the tested conditions. The pH stability profiles of both enzymes were generally comparable across the tested pH range (Figure 2D). During 6 h of incubation, both enzymes retained more than 50% of their initial activity.

These results indicate that the S142F mutation enhanced thermostability while largely maintaining the optimal temperature and pH preferences of PnCLE. This improvement may be associated with changes in local packing and interfacial interactions introduced by the S142F substitution (Nezhad et al., 2023). Additionally, kinetic analysis using *p*-nitrophenyl hexanoate (*p*NPH) as a soluble surrogate substrate was performed to compare the intrinsic catalytic properties of both enzymes. The results revealed that WT PnCLE exhibited a *K*m of 0.087 ± 0.032 mmol L^-1^, a *V*max of 4.90 ± 0.48 μM min^-1^, and a *k*cat of 1.78 ± 0.18 s^-1^. In comparison, the S142F mutant exhibited a lower *K*m of 0.068 ± 0.011 mmol L^-1^, a significantly higher *V*max of 57.98 ± 2.33 μM min^-1^, and a *k*cat of 21.19 ± 0.85 s^-1^ (Supplementary Figure S4), indicating enhanced catalytic performance toward the soluble model substrate. Because *p*NPH hydrolysis does not require solid-surface adsorption, these substantial improvements in *V*max and *k*cat indicate that the S142F mutation improves intrinsic catalytic performance toward the soluble model substrate and may facilitate substrate accommodation within the active site. Therefore, the improved solid PLA degradation efficiency is not solely dependent on enhanced interfacial adsorption, but rather a synergistic result of both enhanced polymer-surface binding and improved intrinsic catalytic properties.

Notably, as observed in the temperature profile, the S142F mutant exhibited a distinct temperature-dependent activity trade-off. Because the temperature profiles demonstrate relative changes within each experimental group, it is visually apparent that S142F exhibits a sharper relative decline at lower temperatures compared to the WT. However, as evidenced by the kinetic parameters, S142F exhibited substantially increased catalytic turnover toward the soluble pNPH substrate at 60 °C. This phenomenon suggests that the introduction of a bulky, hydrophobic phenylalanine at the pocket entrance might alter local conformational flexibility. At lower temperatures, this reduced flexibility may contribute to the conformational dynamics required for efficient substrate entry and catalytic turnover (Campbell et al., 2016). However, as the temperature approaches the glass transition temperature of PLA (∼60 °C), the increased kinetic energy may enhance the flexibility of the enzyme’s binding pocket and increase the mobility of the PLA polymer chains (Lim et al., 2008; Brdlík et al., 2021). Consequently, the potential benefits of increased hydrophobic interactions associated with the S142F mutation may outweigh the initial steric constraints, leading to a significantly higher degradation efficiency at 60 °C. These results suggest that S142F may represent a temperature-adapted variant with improved performance under high-temperature PLA degradation conditions (Mouhoubi et al., 2022).

### 3.3 Enhanced solid PLA film degradation by the S142F mutant

The degradation performance of the S142F mutant toward PLA films was evaluated by weight-loss measurements morphological analysis, and biochemical product quantification. The 36 h degradation assay was performed at 60 °C, corresponding to the optimal temperature determined in this study. To minimize potential effects of thermal inactivation during long-term incubation, the enzyme solution was refreshed every 6 h.

**FIGURE 3:**
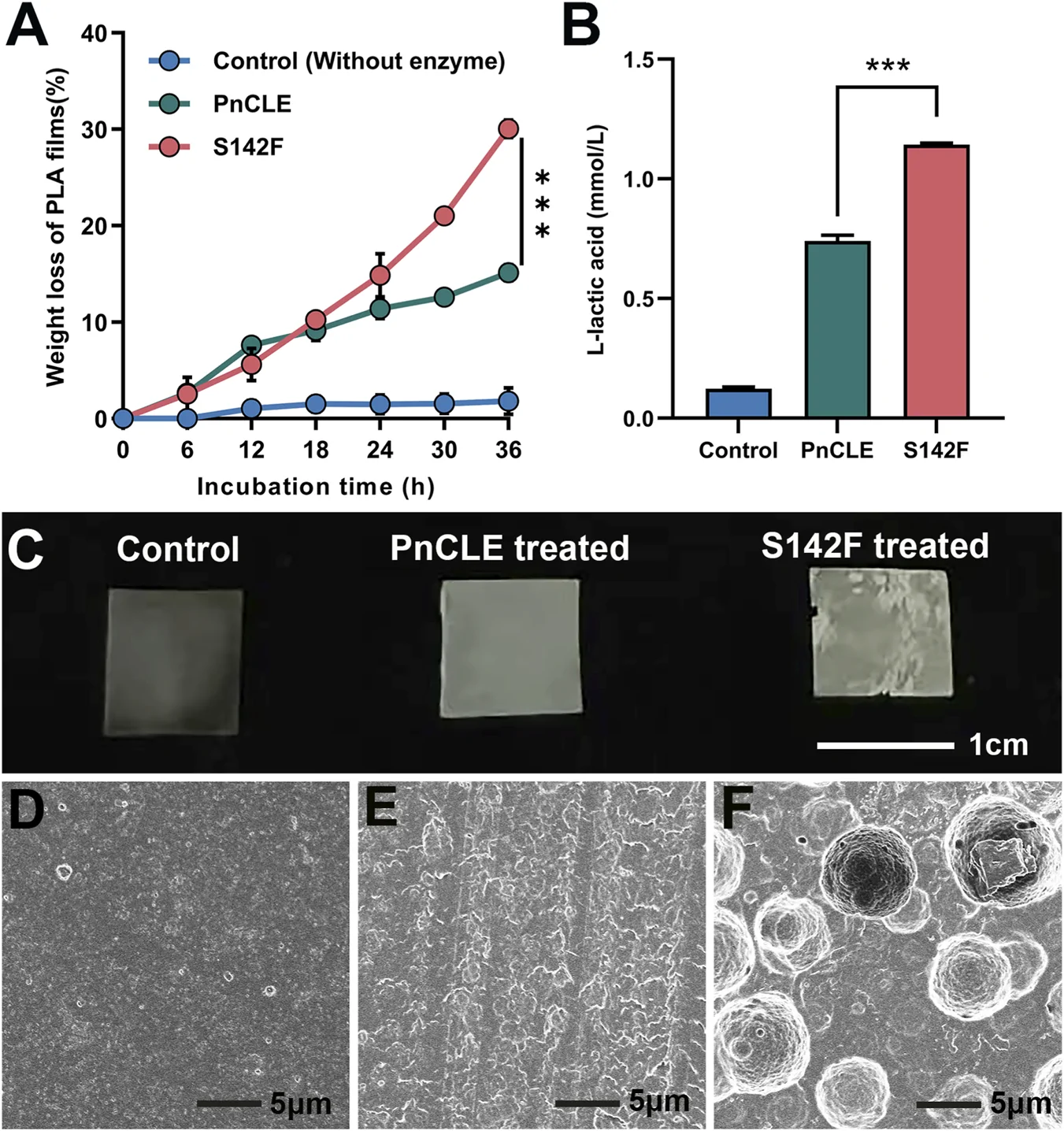
PLA film degradation by WT PnCLE and S142F. Data are presented as mean ± SD, ***p < 0.001. **(A)** Weight loss of PLA films during incubation at 60 °C. **(B)** L-lactic acid released into the supernatant after 36 h of degradation. **(C)** Macroscopic images after 36 h **(D–F)** SEM images of PLA films treated with buffer only (control), WT PnCLE, and S142F, respectively.

As shown in Figure 3A, both WT and S142F exhibited time-dependent degradation of PLA films. After 36 h of incubation, the WT enzyme resulted in a 15.12% weight loss, whereas the S142F mutant achieved 30.25%, representing an approximately two-fold increase (100% enhancement) compared with WT. The S142F mutant consistently showed a higher degradation rate throughout the incubation period. To provide additional biochemical evidence for the enhanced PLA degradation observed from the weight-loss measurements, the released L-lactic acid into the reaction supernatant after 36 h was quantitatively analyzed. Consistent with the weight loss findings, the S142F mutant generated 1.14 mmol L^-1^ of L-lactic acid, representing an approximately 1.58-fold increase compared with WT PnCLE (0.72 mmol L^-1^) (Figure 3B). Macroscopic observations further supported these differences (Figure 3C). The untreated control film remained transparent and intact. The WT-treated film exhibited slight opacity and minor surface deterioration, whereas the S142F-treated film became highly opaque, brittle, and partially fragmented, suggesting more extensive polymer disruption. The whitening phenomenon observed in degraded PLA films is commonly attributed to surface erosion, preferential degradation of amorphous regions, and the formation of microvoids that enhance light scattering (Shalem et al., 2024). Scanning electron microscopy (SEM) provided further qualitative evidence of surface morphological changes. The control film displayed a smooth and uniform surface without visible defects (Figure 3D). The WT-treated film showed mild surface roughening and initial erosion features (Figure 3E), whereas the S142F-treated film exhibited more pronounced surface alterations, including extensive surface erosion, crater-like pits, and cavity formation (Figure 3F). Taken together, the weight-loss measurements, L-lactic acid quantification, and morphological observations consistently support the enhanced PLA degradation performance of the S142F mutant under the tested conditions.

### 3.4 Molecular insights into the enhanced activity of S142F

**FIGURE 4:**
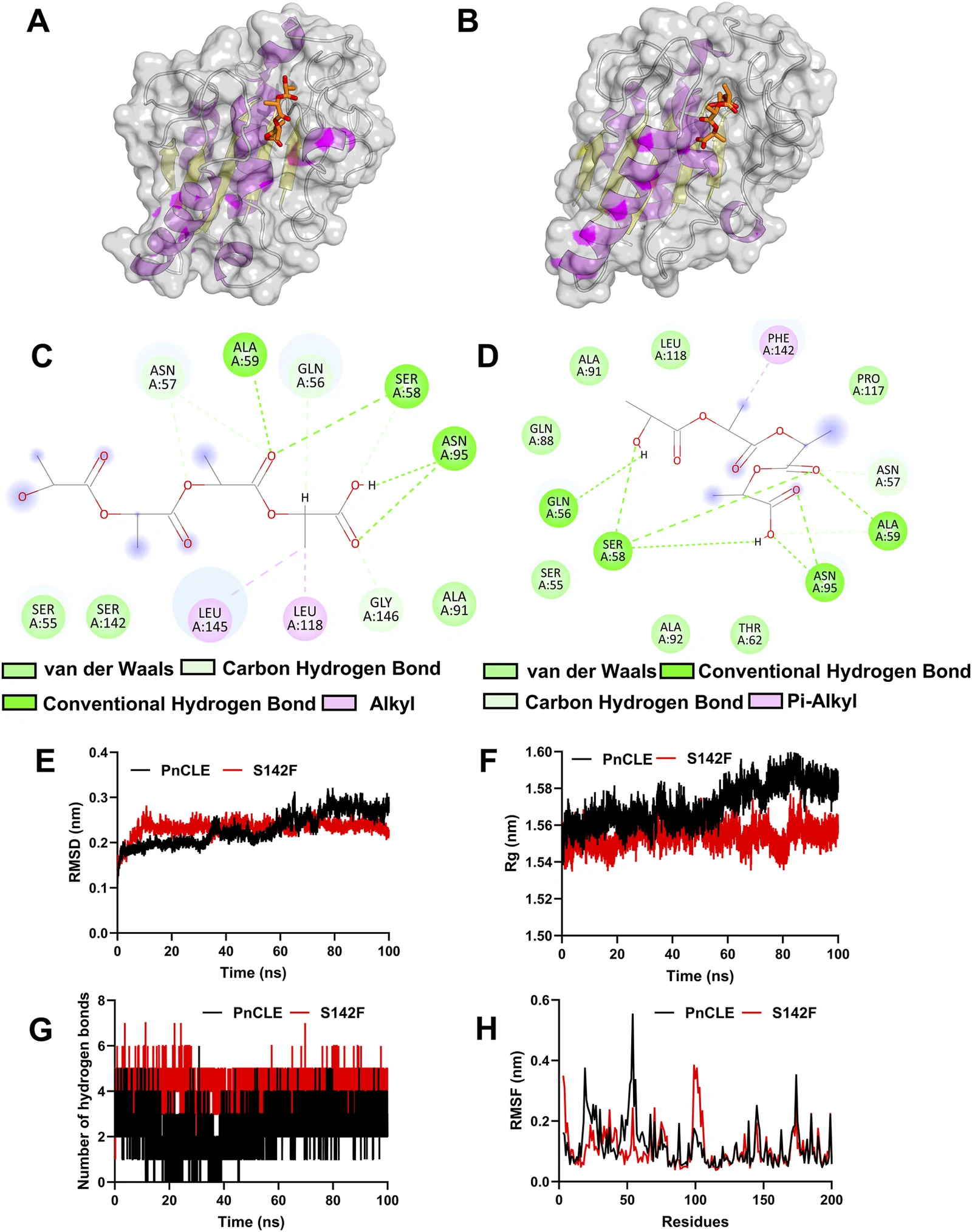
MD simulation analysis of WT PnCLE and S142F mutant in complex with LA_4_. **(A)** Three-dimensional docking model of WT PnCLE–LA_4_ complex; **(B)** Three-dimensional docking model of S142F–LA_4_ complex; **(C)** Two-dimensional interaction map of WT PnCLE–LA_4_ complex; **(D)** Two-dimensional interaction map of S142F–LA_4_ complex; **(E)** Root-mean-square deviation (RMSD) profiles during 100 ns MD simulations; **(F)** Radius of gyration (*R*_g_) profiles of enzyme–substrate complexes; **(G)** Number of hydrogen bonds between enzyme and LA_4_ during simulation; **(H)** Root-mean-square fluctuation (RMSF) of protein residues.

To elucidate the potential molecular basis of the enhanced interfacial adsorption and PLA degradation activity of the S142F mutant, molecular docking and 100 ns molecular dynamics (MD) simulations were performed using AutoDock Vina (Trott and Olson, 2009) and the Amber GAFF force field (Wang et al., 2004), respectively. LA_4_ could be accommodated within the catalytic pocket of both WT PnCLE and S142F, indicating that the overall binding mode was preserved upon mutation. The surface representation further illustrates that LA_4_ is positioned at the substrate-binding region in both enzymes (Figures 4A,B). Two-dimensional interaction analysis revealed distinct binding environments (Figures 4C,D). In WT PnCLE, LA_4_ binding was mainly stabilized by hydrogen bonds, van der Waals forces, and weak hydrophobic interactions, while Ser142 showed limited direct involvement in ligand interaction (Figure 4C). In contrast, in the S142F mutant, the introduction of a phenylalanine residue at position 142 enhanced hydrophobic contacts and introduced additional π–alkyl interactions with LA_4_ (Figure 4D), suggesting improved local hydrophobic compatibility at the pocket entrance.

Although substitution of Ser142 with a bulky phenylalanine residue is expected to increase steric constraints at the substrate-binding pocket entrance, docking analysis indicates that substrate adopts an apparently favorable binding orientation to accommodate the altered pocket geometry. While the MD simulations utilized the LA_4_ tetramer as a simplified substrate model rather than the complete PLA polymer interface, these computational findings, together with the experimental adsorption and degradation results, suggest that the S142F mutation may influence local substrate accommodation and contribute to improved enzyme–polymer interfacial interactions.

MD simulations further supported the potential stabilizing effect of the S142F mutation on the enzyme–substrate complex, which likely underpins the markedly enhanced intrinsic catalytic turnover observed in the kinetic assays. The RMSD profiles indicated that both WT and S142F systems remained stable during the 100 ns simulation (Figure 4E). However, S142F exhibited reduced structural fluctuations and reached equilibrium earlier than WT, suggesting improved dynamic stability. The radius of gyration (*R*_g_) of S142F was slightly lower than that of WT (Figure 4F), indicating a more compact enzyme–substrate conformation. Hydrogen bond analysis showed dynamic fluctuations throughout the simulation (Figure 4G), whereas the S142F complex maintained a higher average number of hydrogen bonds than WT, reflecting more persistent enzyme–substrate interactions. RMSF analysis further revealed reduced flexibility in several loop regions surrounding the substrate-binding pocket in S142F (Figure 4H), indicating stabilization of local conformational dynamics important for substrate binding. This observation highlights the critical role of protein backbone dynamics in modulating enzymatic function (Campbell et al., 2016). The solvent-accessible surface area (SASA) profiles are shown in Supplementary Figure S3; lower SASA values indicate a more compact enzyme–substrate complex.

MM/GBSA calculations further supported the potentially enhanced binding interactions of S142F (Table 1). The total binding free energy decreased from −23.74 ± 1.69 kcal mol^-1^ (WT) to −30.09 ± 1.40 kcal mol^-1^ (S142F), indicating a more favorable binding interaction under identical simulation conditions. Energy decomposition analysis showed that this improvement was mainly attributed to stronger van der Waals interactions (−24.33 vs. −20.08 kcal mol^-1^) and electrostatic contributions (−29.99 vs. −23.51 kcal mol^-1^), consistent with the increased hydrophobic contacts observed in docking analysis. This stronger thermodynamic binding affinity toward the LA_4_ oligomer provides additional molecular insights into the enhanced substrate interactions associated with the improved catalytic performance. It should be noted that MM/GBSA values are used for comparative rather than absolute binding affinity evaluation.

**TABLE 1:** MM/GBSA binding free energy components of WT PnCLE and S142F mutant in complex with LA_4_.

Contribution components	WT (kcal·mol^-1^)	S142F (kcal·mol^-1^)
*Δ*_VDWAALS_	−20.08 ± 0.39	−24.33 ± 0.11
*ΔE*_elec_	−23.51 ± 1.63	−29.99 ± 1.26
*ΔE*_GB_	22.90 ± 0.17	28.33 ± 0.78
*ΔE*_surf_	−3.06 ± 0.02	−4.09 ± 0.17
*ΔG*_gas_	−43.59 ± 1.68	−54.32 ± 1.26
*ΔG*_solvation_	19.85 ± 0.17	24.24 ± 0.80
*Δ*_Total_	−23.74 ± 1.69	−30.09 ± 1.40

**FIGURE 5:**
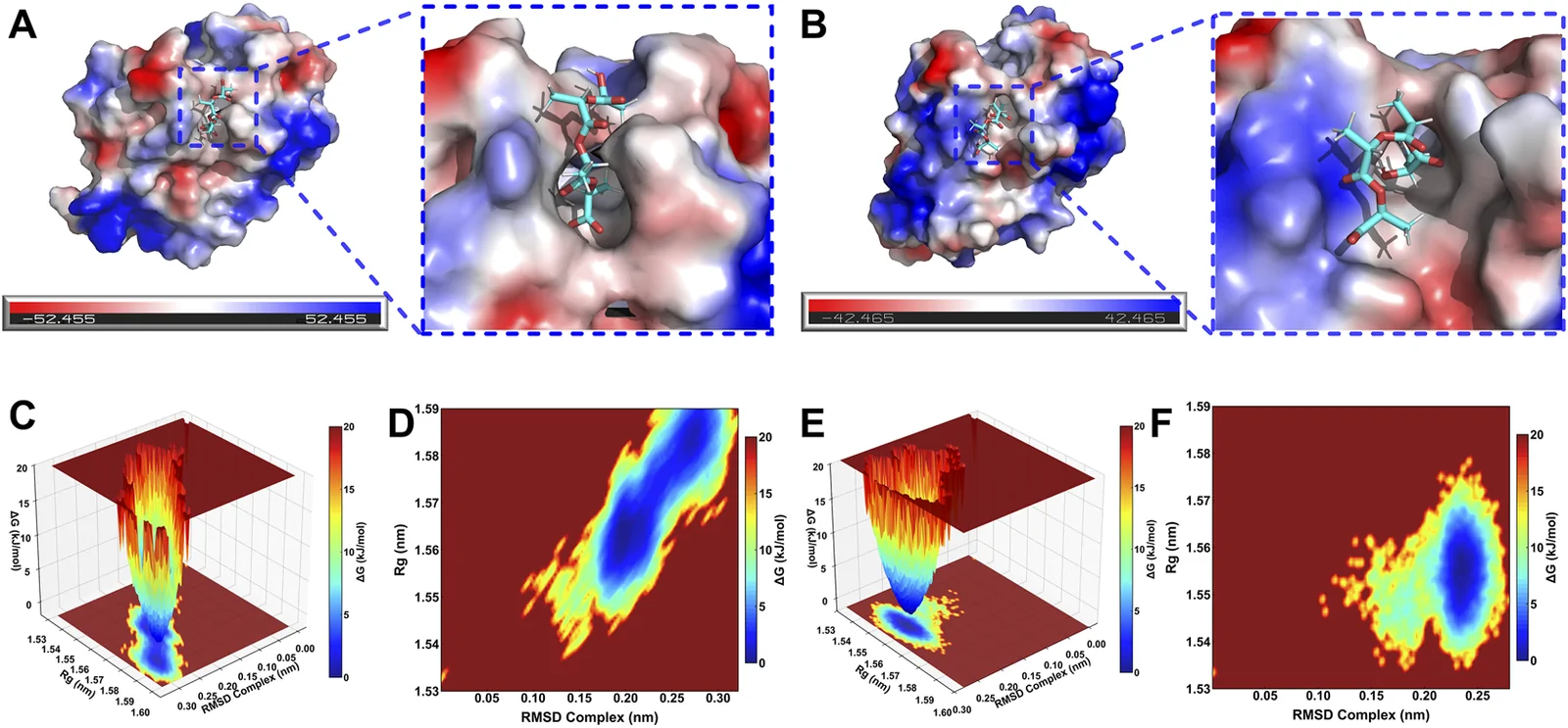
Surface electrostatic potential and free energy landscape analysis of WT PnCLE and S142F mutant. **(A)** Surface electrostatic potential of WT PnCLE; **(B)** Surface electrostatic potential of the S142F mutant; **(C)** Three-dimensional free energy landscape of the WT–LA_4_ complex derived from molecular dynamics simulations; **(D)** Two-dimensional free energy landscape of the WT–LA_4_ complex (RMSD-*R*_g_ space); **(E)** Three-dimensional free energy landscape of the S142F–LA_4_ complex derived from molecular dynamics simulations; **(F)** Two-dimensional free energy landscape of the S142F–LA_4_ complex (RMSD-*Rg* space).

Surface electrostatic potential analysis revealed that the S142F mutation altered the local physicochemical environment near the entrance of the substrate-binding pocket (Figures 5A,B). Compared with WT, S142F exhibited a less polar and more hydrophobic surface around position 142, consistent with substitution of serine by phenylalanine. Given the hydrophobic nature of PLA, this local surface remodeling may facilitate enzyme adsorption onto the polymer surface and contribute to improved enzyme–polymer interfacial recognition. Free energy landscape (FEL) analysis further showed that the S142F–LA_4_ complex sampled a more concentrated low-energy basin than the WT system (Figures 5C–F), indicating a more stable substrate-bound conformational state during simulation.

**FIGURE 6:**
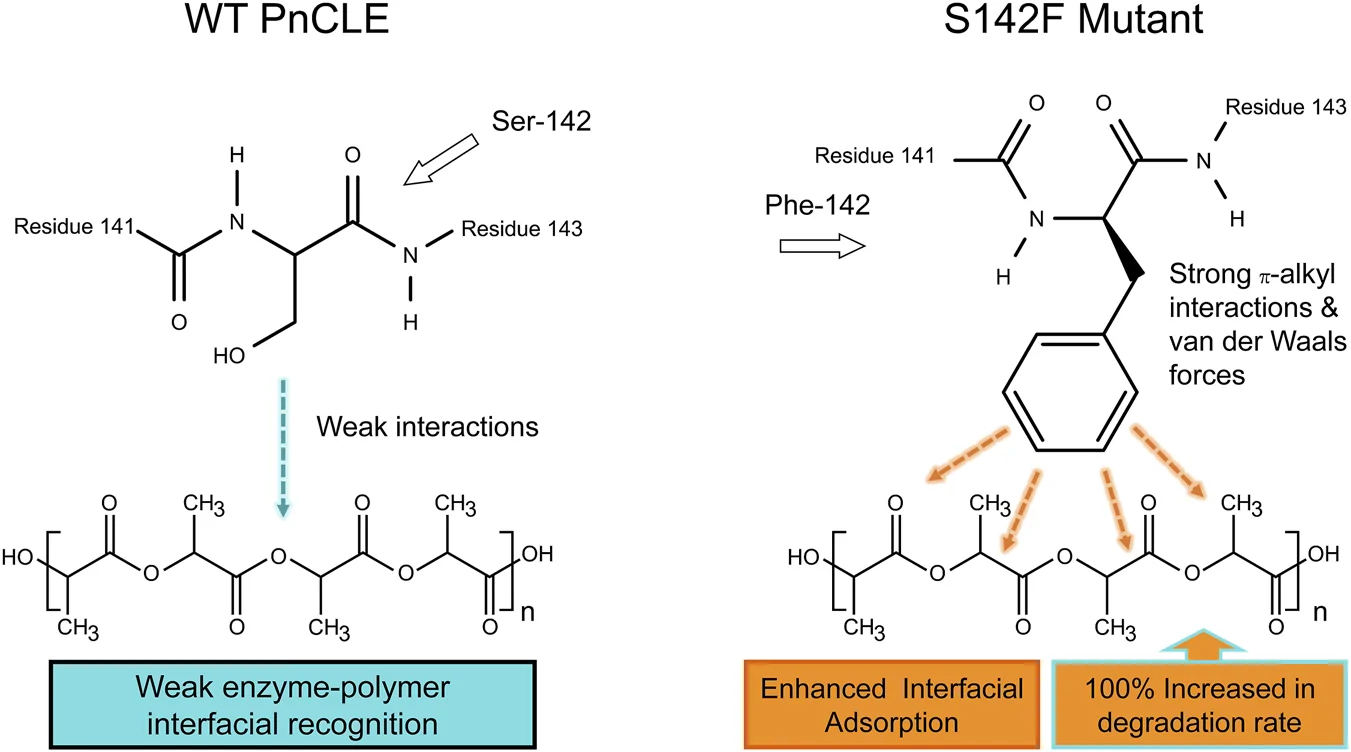
Proposed mechanism of enhanced PLA degradation by WT PnCLE and S142F mutant via gatekeeper residue engineering at the substrate-binding pocket entrance.

Based on these results, a proposed model describing the possible contribution of S142F to enhanced PLA degradation is proposed in Figure 6. In WT PnCLE, the polar Ser142 residue located at the entrance of the substrate-binding pocket may provide limited hydrophobic contacts with PLA surfaces. Upon substitution with phenylalanine, the aromatic side chain increases local hydrophobicity at the pocket entrance and may introduce additional non-covalent interactions with PLA chains. This structural remodeling may promote enzyme–substrate interfacial adsorption, stabilize the enzyme–substrate complex, and promote a more favorable binding conformation during PLA hydrolysis. Accordingly, the improved PLA film degradation performance of S142F may be associated with the synergistic effects of enhanced interfacial adsorption, improved intrinsic catalytic efficiency, and improved conformational stability. These results suggest that engineering substrate-binding regions at pocket entrances may represent a potential strategy for modulating enzyme–polymer interfacial interactions and improve the degradation efficiency of PLA-degrading cutinases.

### 3.5 Benchmarking and industrial implications of the S142F mutation in PLA degradation

To evaluate the performance of the S142F mutant, its PLA degradation efficiency was compared with several well-characterized wild-type and engineered hydrolases reported in the literature (Table 2). Across polyester-degrading systems, early benchmark enzymes such as Proteinase K (Williams, 1981; Hajighasemi et al., 2016) and *Humicola insolens* cutinase (HiC) (Su et al., 2020) have mainly served as reference catalysts under controlled conditions rather than fully optimized industrial biocatalysts. Early protein engineering strategies predominantly focused on enhancing global structural stability, such as the introduction of artificial disulfide bridges into LCC, which significantly improved both thermal stability and overall polyester hydrolysis activity (Tournier et al., 2020). In contrast, more recent efforts have increasingly shifted toward addressing the intrinsic limitations associated with solid-state polymer degradation.

**TABLE 2:** Comparison of PLA and polyester degradation performance of wild-type and engineered hydrolases reported in previous studies.

Enzyme source	Mutation(s)/Variant	Target polymer	Reaction conditions	Degradation performance	Key engineering strategy/Structural feature	References
*Papiliotrema nemorosa* (PnCLE)	S142F	PLA film	60 °C, 36 h	30.25% weight loss	Hydrophobization of the gatekeeper residue at the pocket entrance to enhance enzyme–polymer interfacial interactions and catalytic performance	This study
Metagenomic compost leaf/branch cutinase (LCC)	ICCG variant	PET microparticles	72 °C, 9.3–10.5 h	>90% depolymerization efficiency	Rational active-site architecture tuning combined with calcium-mimicking disulfide bond stabilization	Tournier et al., 2020
*Aspergillus nidulans* (ANCUT1)	Wild-type	PLA packaging	45 °C, 120 h	45.96% weight loss	Highly versatile fungal cutinase with broad structural adaptability to synthetic polyesters	Alvarado et al., 2024
*Saccharomonospora viridis* (Cut190 variant)	S226P/R228S/D250C–E296C	PET film	70 °C, 48 h	>30% weight loss	Disulfide loop rigidification to simulate Ca^2+^ binding networks and suppress thermal fluctuation	Kawai et al., 2014; Miyakawa et al., 2015
*Humicola insolens* cutinase (HiC)	Pocket entrance variants	Soluble polyacrylates	40 °C, 24 h	Improved ester bond cleavage	Modification of gatekeeper residues to reduce steric hindrance at the substrate-binding pocket entrance	Su et al., 2020
*Humicola insolens* cutinase (HiC)	Wild-type	Mixed PET/cotton	55 °C, 48 h	Quantitative ester conversion	Deployment of industrial-grade cutinase for high-moisture solid-state interfacial enzymatic hydrolysis	Kaabel et al., 2022
*Fusarium solani* cutinase	Wild-type/Recombinant	PBS film	50 °C, 24 h	16.8% weight loss	Comprehensive characterization of aliphatic polyester degradation and ester-bond cleavage kinetics	Hu et al., 2016
*Tritirachium album* (Proteinase K)	Wild-type	PLA solid matrix	37 °C–50 °C, 24 h	High endo-cleavage activity	Traditional reference serine protease acting as a standard benchmark for enzymatic PLA cleavage	Williams, 1981; Hajighasemi et al., 2016

Representative advances include LCC-ICCG, which enabled near-complete PET depolymerization within 10–15 h at 65 °C through multi-site active-site engineering (Tournier et al., 2020), and Cut190 variants, which enhanced PET hydrolysis via loop optimization and calcium-binding stabilization (Kawai et al., 2014; Miyakawa et al., 2015). These systems highlight that high-efficiency depolymerization typically relies on coordinated multi-site or global structural modifications. Beyond PET systems, cutinases have been applied to diverse polyester substrates under different operational regimes. HiC has been directly used for mixed PET/cotton textile hydrolysis under industrially relevant conditions (Kaabel et al., 2022), while engineered variants targeting the substrate-binding region have improved activity toward polyacrylates (Su et al., 2020). For aliphatic polyesters, ANCUT1 and *Fusarium solani* cutinase (FsC) achieved 45.96% PLA degradation and 16.8% PBS hydrolysis under optimized conditions, respectively (Hu et al., 2016; Alvarado et al., 2024). Collectively, these studies indicate that performance enhancement in polyester-degrading enzymes has predominantly relied on multi-site engineering or global structural optimization strategies.

Against this background, the engineered S142F mutant achieved 30.25% PLA film weight loss within 36 h at 60 °C, demonstrating efficient degradation under conditions relevant to industrial composting. In contrast to multi-site engineered systems such as LCC-ICCG, the present variant achieves competitive PLA hydrolysis performance through a single-point mutation at the substrate-binding pocket entrance. This result suggests that modulation of enzyme–polymer interfacial recognition may serve as a complementary strategy to catalytic-core optimization for solid-state polyester degradation.

From a structural perspective, the success of this rational design was enabled by the accuracy of AlphaFold3-predicted structures (Abramson et al., 2024). Rather than relying on close homologs as required in conventional homology modeling, AlphaFold3 provides high-confidence structural predictions that facilitate the identification of candidate residues for further engineering rather than directly defining functional determinants. This capability may accelerate rational enzyme design by enabling exploration of substrate-binding regions involved in enzyme–polymer interactions. Taken together, these results suggest that interfacial engineering at substrate-binding regions represents a promising approach for improving PLA-degrading enzyme performance.

The primary objective of this study was to evaluate the feasibility of substrate-binding region engineering to enhance enzyme–polymer interfacial adsorption and overall degradation efficiency. While the significant weight loss, SEM observations, and L-lactic acid monomer quantification provide complementary macroscopic, microscopic, and biochemical evidence supporting the enhanced PLA film degradation performance of the S142F variant, these methods do not fully resolve the exact biochemical depolymerization pathway. Further studies incorporating HPLC or LC-MS will be necessary to profile released intermediate oligomers and characterize changes in polymer molecular weight by GPC and crystallinity by DSC or XRD before and after enzymatic treatment (Karamanlioglu et al., 2017; Shalem et al., 2024).

## 4 Conclusion

In this study, a cutinase from *Papiliotrema nemorosa* (PnCLE) was successfully expressed and characterized in *Escherichia coli*. PnCLE exhibited an optimal catalytic temperature of 60 °C, which is higher than that reported for native Cr14CLE and is well aligned with the temperature range of industrial composting (55 °C–60 °C). Through rational engineering of the substrate-binding region surrounding the gatekeeper residue Ser142, the S142F mutant was obtained, showing significantly improved PLA degradation performance compared with the wild-type enzyme. This improvement likely resulted from the synergistic contribution of enhanced interfacial adsorption and improved intrinsic catalytic properties, leading to increased emulsion hydrolysis activity and higher PLA film degradation efficiency. Under industrially relevant conditions (36 h at 60 °C with periodic enzyme replenishment), the S142F mutant achieved a maximum PLA film weight loss of 30.25%. This macroscopic disruption was biochemically validated by a 1.58-fold increase in the release of L-lactic acid monomer compared to the wild-type enzyme. Molecular dynamics simulations suggested that the S142F mutation increased local hydrophobicity at the substrate-binding pocket entrance, which may contribute to enhanced enzyme–substrate interactions and a more stable binding conformation. These results suggest that engineering substrate-binding regions at the substrate-binding pocket entrance may represent a potential strategy for modulating enzyme–polymer interfacial interactions and optimizing active-site dynamics, thereby enhancing PLA degradation efficiency. The S142F variant provides a promising biocatalyst for PLA biodegradation under high-temperature degradation conditions. Future work will focus on further improving catalytic performance through combinatorial mutagenesis and elucidating the mechanistic basis of the observed differences in optimal temperature.

## Data Availability

The original contributions presented in the study are included in the article/Supplementary Material, further inquiries can be directed to the corresponding author.

## References

[B1] Abramson J. Adler J. Dunger J. Evans R. Green T. Pritzel A. (2024). Accurate structure prediction of biomolecular interactions with AlphaFold 3. Nature 630, 493–500. 10.1038/s41586-024-07487-w 38718835 PMC11168924

[B2] Alvarado E. Castro R. Castro-Rodríguez J. A. Navarro A. Farrés A. (2024). Poly(lactic acid) degradation by recombinant cutinases from aspergillus nidulans. Polymers 16, 1994. 10.3390/polym16141994 39065311 PMC11281152

[B3] Amobonye A. Bhagwat P. Singh S. Pillai S. (2021). Plastic biodegradation: frontline microbes and their enzymes. Sci. Total Environ. 759, 143536. 10.1016/j.scitotenv.2020.143536 33190901

[B4] Arunrattanamook N. Mhuantong W. Paemanee A. Reamtong O. Hararak B. Champreda V. (2023). Identification of a plastic-degrading enzyme from cryptococcus nemorosus and its use in self-degradable plastics. Appl. Microbiol. Biotechnol. 107, 7439–7450. 10.1007/s00253-023-12816-6 37801098

[B5] Austin H. P. Allen M. D. Donohoe B. S. Rorrer N. A. Kearns F. L. Silveira R. L. (2018). Characterization and engineering of a plastic-degrading aromatic polyesterase. Proc. Natl. Acad. Sci. U.S.A. 115, E4350–E4357. 10.1073/pnas.1718804115 29666242 PMC5948967

[B6] Brdlík P. Borůvka M. Běhálek L. Lenfeld P. (2021). Biodegradation of poly(lactic acid) biocomposites under controlled composting conditions and freshwater biotope. Polymers 13, 594. 10.3390/polym13040594 33669420 PMC7920484

[B7] Campbell E. Kaltenbach M. Correy G. J. Carr P. D. Porebski B. T. Livingstone E. K. (2016). The role of protein dynamics in the evolution of new enzyme function. Nat. Chem. Biol. 12, 944–950. 10.1038/nchembio.2175 27618189

[B8] Dąbrowska G. B. Garstecka Z. Olewnik-Kruszkowska E. Szczepańska G. Ostrowski M. Mierek-Adamska A. (2021). Comparative study of structural changes of polylactide and poly(ethylene terephthalate) in the presence of trichoderma viride. Int. J. Mol. Sci. 22, 3491. 10.3390/ijms22073491 33800567 PMC8038068

[B9] Farah S. Anderson D. G. Langer R. (2016). Physical and mechanical properties of PLA, and their functions in widespread applications — a comprehensive review. Adv. Drug Deliv. Rev. 107, 367–392. 10.1016/j.addr.2016.06.012 27356150

[B10] Genheden S. Ryde U. (2015). The MM/PBSA and MM/GBSA methods to estimate ligand-binding affinities. Expert Opin. Drug Discov. 10, 449–461. 10.1517/17460441.2015.1032936 25835573 PMC4487606

[B11] Geyer R. Jambeck J. R. Law K. L. (2017). Production, use, and fate of all plastics ever made. Sci. Adv. 3, e1700782. 10.1126/sciadv.1700782 28776036 PMC5517107

[B12] Hajighasemi M. Nocek B. P. Tchigvintsev A. Brown G. Flick R. Xu X. (2016). Biochemical and structural insights into enzymatic depolymerization of polylactic acid and other polyesters by microbial carboxylesterases. Biomacromolecules 17, 2027–2039. 10.1021/acs.biomac.6b00223 27087107 PMC6886529

[B13] Hajighasemi M. Tchigvintsev A. Nocek B. Flick R. Popovic A. Hai T. (2018). Screening and characterization of novel polyesterases from environmental metagenomes with high hydrolytic activity against synthetic polyesters. Environ. Sci. & Technol. 52, 12388–12401. 10.1021/acs.est.8b04252 30284819 PMC12447631

[B14] Hu X. Gao Z. Wang Z. Su T. Yang L. Li P. (2016). Enzymatic degradation of poly(butylene succinate) by cutinase cloned from *Fusarium solani* . Polym. Degrad. Stab. 134, 211–219. 10.1016/j.polymdegradstab.2016.10.012

[B15] Joo S. Cho I. J. Seo H. Son H. F. Sagong H.-Y. Shin T. J. (2018). Structural insight into molecular mechanism of poly(ethylene terephthalate) degradation. Nat. Commun. 9, 382. 10.1038/s41467-018-02881-1 29374183 PMC5785972

[B16] Kaabel S. Arciszewski J. Borchers T. H. Therien J. P. D. Friščić T. Auclair K. (2022). Solid‐state enzymatic hydrolysis of mixed PET/cotton textiles. ChemSusChem 16, e202201613. 10.1002/cssc.202201613 36165763

[B17] Karamanlioglu M. Preziosi R. Robson G. D. (2017). Abiotic and biotic environmental degradation of the bioplastic polymer poly(lactic acid): a review. Polym. Degrad. Stab. 137, 122–130. 10.1016/j.polymdegradstab.2017.01.009

[B18] Kawai F. Oda M. Tamashiro T. Waku T. Tanaka N. Yamamoto M. (2014). A novel Ca^2+^-activated, thermostabilized polyesterase capable of hydrolyzing polyethylene terephthalate from saccharomonospora viridis AHK190. Appl. Microbiol. Biotechnol. 98, 10053–10064. 10.1007/s00253-014-5860-y 24929560

[B19] Lim L.-T. Auras R. Rubino M. (2008). Processing technologies for poly(lactic acid). Prog. Polym. Sci. 33, 820–852. 10.1016/j.progpolymsci.2008.05.004

[B20] Masaki K. Kamini N. R. Ikeda H. Iefuji H. (2005). Cutinase-like enzyme from the yeast cryptococcus sp. Strain S-2 hydrolyzes polylactic acid and other biodegradable plastics. Appl. Environ. Microbiol. 71, 7548–7550. 10.1128/AEM.71.11.7548-7550.2005 16269800 PMC1287645

[B21] Miyakawa T. Mizushima H. Ohtsuka J. Oda M. Kawai F. Tanokura M. (2015). Structural basis for the Ca^2+^-enhanced thermostability and activity of PET-degrading cutinase-like enzyme from *Saccharomonospora viridis* AHK190. Appl. Microbiol. Biotechnol. 99, 4297–4307. 10.1007/s00253-014-6272-8 25492421

[B22] Mouhoubi R. Lasschuijt M. Ramon Carrasco S. Gojzewski H. Wurm F. R. (2022). End-of-life biodegradation? How to assess the composting of polyesters in the lab and the field. Waste Manag. 154, 36–48. 10.1016/j.wasman.2022.09.025 36209717

[B23] Naser A. Z. Deiab I. Darras B. M. (2021). Poly(lactic acid) (PLA) and polyhydroxyalkanoates (PHAs), green alternatives to petroleum-based plastics: a review. RSC Adv. 11, 17151–17196. 10.1039/d1ra02390j 35479695 PMC9033233

[B24] Nezhad N. G. Rahman R. N. Z. R. A. Normi Y. M. Oslan S. N. Shariff F. M. Leow T. C. (2023). Recent advances in simultaneous thermostability-activity improvement of industrial enzymes through structure modification. Int. J. Biol. Macromol. 232, 123440. 10.1016/j.ijbiomac.2023.123440 36708895

[B25] Ribitsch D. Herrero Acero E. Greimel K. Dellacher A. Zitzenbacher S. Marold A. (2012). A new esterase from *Thermobifida halotolerans* hydrolyses polyethylene terephthalate (PET) and polylactic acid (PLA). Polymers. 4, 617–629. 10.3390/polym4010617

[B26] Ribitsch D. Yebra A. O. Zitzenbacher S. Wu J. Et A. Steinkellner G. (2013). Fusion of binding domains to *Thermobifida cellulosilytica* cutinase to tune sorption characteristics and enhancing PET hydrolysis. Biomacromolecules 14, 1769–1776. 10.1021/bm400140u 23718548

[B27] Shah A. A. Hasan F. Hameed A. Ahmed S. (2008). Biological degradation of plastics: a comprehensive review. Biotechnol. Adv. 26, 246–265. 10.1016/j.biotechadv.2007.12.005 18337047

[B28] Shalem A. Yehezkeli O. Fishman A. (2024). Enzymatic degradation of polylactic acid (PLA). Appl. Microbiol. Biotechnol. 108, 413. 10.1007/s00253-024-13212-4 38985324 PMC11236915

[B29] Shin N. Oh J. Kim S. Lee Y. Shin Y. Choi S. (2024). Dual application of *p* -nitrophenol alkanoate-based assay for soil selection and screening of microbial strains for bioplastic degradation. J. Microbiol. Biotechnol. 34, 1530–1543. 10.4014/jmb.2403.03013 38973389 PMC11294652

[B30] Singh N. Walker T. R. (2024). Plastic recycling: a panacea or environmental pollution problem. Npj Mater. Sustain. 2, 17. 10.1038/s44296-024-00024-w 39114578 PMC11304528

[B31] Su L. Hong R. Kong D. Wu J. (2020). Enhanced activity towards polyacrylates and poly(vinyl acetate) by site-directed mutagenesis of *humicola insolens* cutinase. Int. J. Biol. Macromol. 162, 1752–1759. 10.1016/j.ijbiomac.2020.07.261 32771512

[B32] Tokiwa Y. Calabia B. P. (2006). Biodegradability and biodegradation of poly(lactide). Appl. Microbiol. Biotechnol. 72, 244–251. 10.1007/s00253-006-0488-1 16823551

[B33] Tournier V. Topham C. M. Gilles A. David B. Folgoas C. Moya-Leclair E. (2020). An engineered PET depolymerase to break down and recycle plastic bottles. Nature 580, 216–219. 10.1038/s41586-020-2149-4 32269349

[B34] Trott O. Olson A. J. (2009). AutoDock vina: improving the speed and accuracy of docking with a new scoring function, efficient optimization, and multithreading. J. Comput. Chem. 31, 455–461. 10.1002/jcc.21334 19499576 PMC3041641

[B35] Wang J. Wolf R. M. Caldwell J. W. Kollman P. A. Case D. A. (2004). Development and testing of a general amber force field. J. Comput. Chem. 25, 1157–1174. 10.1002/jcc.20035 15116359

[B36] Wang G. X. Huang D. Ji J.-H. Völker C. Wurm F. R. (2020). Seawater‐degradable polymers—fighting the marine plastic pollution. Adv. Sci. 8, 2001121. 10.1002/advs.202001121 33437568 PMC7788598

[B37] Wei R. Tiso T. Bertling J. O’Connor K. Blank L. M. Bornscheuer U. T. (2020). Possibilities and limitations of biotechnological plastic degradation and recycling. Nat. Catal. 3, 867–871. 10.1038/s41929-020-00521-w

[B38] Williams D. F. (1981). Enzymic hydrolysis of polylactic acid. Eng. Med. 10, 5–7. 10.1243/emed_jour_1981_010_004_02

[B39] Xu A. Zhou J. Blank L. M. Jiang M. (2023). Future focuses of enzymatic plastic degradation. Trends Microbiol. 31, 668–671. 10.1016/j.tim.2023.04.002 37121829

[B40] Yang Y. Min J. Xue T. Jiang P. Liu X. Peng R. (2023). Complete bio-degradation of poly(butylene adipate-co-terephthalate) via engineered cutinases. Nat. Commun. 14, 1645. 10.1038/s41467-023-37374-3 36964144 PMC10039075

