# Supplementary material for: Structure-guided engineering of a gatekeeper residue enhances polylactic acid depolymerization by a cutinase from Papiliotrema nemorosa
Source: Front Bioeng Biotechnol. 2026 Sep 10;14:1936157. doi: 10.3389/fbioe.2026.1936157 (PMC13601345; doi:10.3389/fbioe.2026.1936157)
Supplement: Supplementary file 1 [file Supplementaryfile1.docx]

Supplementary Material

Supplementary Figure 1 SDS-PAGE of recombinant PnCLE. M, molecular weight marker; lane 1, whole-cell lysate of uninduced *E. coli* BL21(DE3)/pET-22b(+)-PnCLE; lane 2, whole-cell lysate of IPTG-induced *E. coli* BL21(DE3)/pET-22b(+)-PnCLE; lane 3, soluble fraction after sonication; lane 4, insoluble fraction after sonication; lane 5, purified PnCLE. The gel concentration was 12%.

Supplementary Figure 2 Time-dependent decrease in PLA emulsion turbidity (OD_630_) during enzymatic degradation by WT PnCLE and mutant variants. The control represents buffer without enzyme.

Supplementary Figure 3 Time evolution of solvent-accessible surface area (SASA) during 100 ns molecular dynamics simulations for WT and S142F enzyme-substrate complexes.

Supplementary Figure 4 Kinetic analysis of WT PnCLE and the S142F mutant using *p*-nitrophenyl hexanoate (*p*NPH) as the substrate. (A) Michaelis-Menten plots showing the reaction velocity versus substrate concentration for both enzymes. (B) Lineweaver-Burk double-reciprocal plots used to determine the kinetic parameters (*K*_m_ and *V*_max_).

Supplementary Table 1 Primer sequences for site-directed mutagenesis

| **Mutants** | **Primer** | **Sequence (5’ to 3’)** |
| --- | --- | --- |
| P117Y | F | CTGATTGGTAATTACCTGCATAAAGCCGGT |
|  | R | ACCGGCTTTATGCAGGTAATTACCAATCAG |
| I141W | F | AATACCCGCGGTTGGAGCGCCGGTCTG |
|  | R | CAGACCGGCGC TCCAACCGCGGGTATT |
| S142W | F | ACCCGCGGTATT TGGGCCGGTCTGGGT |
|  | R | ACCCAGACCGGCCCAAATACCGCGGGT |
| S142Y | F | ACCCGCGGTATTTATGCCGGTCTGGGT |
|  | R | ACCCAGACCGGCATAAATACCGCGGGT |
| S142F | F | ACCCGCGGTATTTTTGCCGGTCTGGGT |
|  | R | ACCCAGACCGGCAAAAATACCGCGGGT |
| S142V | F | ACCCGCGGTATTGTAGCCGGTCTGGGT |
|  | R | ACCCAGACCGGCTACAATACCGCGGGT |
| S142D | F | ACCCGCGGTATTGACGCCGGTCTGGGT |
|  | R | ACCCAGACCGGCGTCAATACCGCGGGT |
| S142C | F | ACCCGCGGTATTTGTGCCGGTCTGGGT |
|  | R | ACCCAGACCGGCACAAATACCGCGGGT |
| S142A | F | ACCCGCGGTATTGCAGCCGGTCTGGGT |
|  | R | ACCCAGACCGGCTGCAATACCGCGGGT |

| **Mutants** | **Primer** | **Sequence (5’ to 3’)** |
| --- | --- | --- |
| L145Y | F | GGTATTAGCGCCGGTTACGGTGGTGGTATTCCG |
|  | R | CGGAATACCACCACCGTAACCGGCGCTAATACC |
| L145K | F | GGTATTAGCGCCGGTAAGGGTGGTGGTATTCCG |
|  | R | CGGAATACCACCACCCTTACCGGCGCTAATACC |

Supplementary Table 2 PCR reaction conditions for site-directed mutagenesis

| **Step** | **Cycle(s)** | **Temperature** | **Time** | **Stage** |
| --- | --- | --- | --- | --- |
| 1 | 1 | 95 ℃ | 3min | Initial denaturation |
| 2 | 20 | 95 ℃ | 30sec | Denaturation |
|  |  | 55 ℃ | 30sec | Annealing |
|  |  | 68 ℃ | 3min | Extension |
| 3 | 1 | 68 ℃ | 10min | Final extension |
| 4 | 1 | 4 ℃ | - | Temporary storage |

Supplementary Table 3 Alanine scanning results

| **No.** | **Mutation** | **Mutation Energy(kcal·mol^-1^)** | **Effect of Mutation** |
| --- | --- | --- | --- |
| 1 | A: GLY146>ALA | 1.26 | DESTABILIZING |
| 2 | A: ASN57>ALA | 1.23 | DESTABILIZING |
| 3 | A: ASN95>ALA | 0.98 | DESTABILIZING |
| 4 | A: ALA60>ALA | 0.97 | DESTABILIZING |
| 5 | A: LEU118>ALA | 0.72 | DESTABILIZING |
| 6 | A: GLN88>ALA | 0.59 | DESTABILIZING |
| 7 | A: GLN56>ALA | 0.57 | DESTABILIZING |
| 8 | A: THR62>ALA | 0.54 | DESTABILIZING |
| 9 | A: ALA91>ALA | 0.28 | NEUTRAL |
| 10 | A: ILE141>ALA | 0.23 | NEUTRAL |
| 11 | A: SER58>ALA | 0.16 | NEUTRAL |
| 12 | A: PRO117>ALA | 0.1 | NEUTRAL |
| 13 | A: LEU145>ALA | 0.07 | NEUTRAL |
| 14 | A: ALA92>ALA | 0.02 | NEUTRAL |
| 15 | A: ALA59>ALA | 0.01 | NEUTRAL |
| 16 | A: GLY61>ALA | -0.02 | NEUTRAL |
| 17 | A: SER142>ALA | -0.2 | NEUTRAL |

Supplementary Table 4 Virtual saturation mutagenesis results

| **No.** | **Mutation** | **Mutation Energy(kcal·mol^-1^)** | **Effect** |
| --- | --- | --- | --- |
| 1 | A:SER142>TRP | -1.23 | STABILIZING |
| 2 | A:SER142>TYR | -1.03 | STABILIZING |
| 3 | A:SER142>PHE | -0.96 | STABILIZING |
| 4 | A:ILE141>TRP | -0.77 | STABILIZING |
| 5 | A:LEU145>TYR | -0.63 | STABILIZING |
| 6 | A:LEU145>LYS | -0.55 | STABILIZING |
| 7 | A:SER142>VAL | -0.53 | STABILIZING |
| 8 | A:PRO117>TYR | -0.52 | STABILIZING |
